# Evaluation of a Young Dog for Escalating Aggression Toward Both Familiar and Unfamiliar People

**DOI:** 10.1155/crve/9804544

**Published:** 2026-05-27

**Authors:** Lynn Honeckman

**Affiliations:** ^1^ Veterinary Behavior Solutions, Orlando, Florida, USA

## Abstract

This case report describes the evaluation and successful multimodal treatment of severe, early‐onset aggression in a juvenile dog. A 9‐month‐old spayed female Australian Shepherd presented with escalating aggressive behaviors toward familiar and unfamiliar people, including multiple bite incidents resulting in human injury. Aggression was observed across numerous contexts, including handling, play, resource guarding, visitor interactions, and encounters with people and dogs outside the home. Significant risk factors included early removal from the dam at 6 weeks of age, minimal early socialization, a history of chronic illness during early development, and later‐discovered aggressive traits in both parents. Comprehensive medical evaluation, including repeated laboratory testing and neurological assessment, revealed no underlying medical abnormalities. Behavioral diagnoses included generalized anxiety, impulse‐control aggression, fear‐related aggression, possessive aggression, and socialization deficits. Prior to referral, behavioral issues were minimized by outside advisors, and no psychopharmacologic intervention had been attempted due to the dog′s young age. By 9 months, the family was considering rehoming or behavioral euthanasia due to safety concerns and breakdown of the human–animal bond. Treatment consisted of an integrated plan incorporating environmental management, strict safety protocols, positive reinforcement–based behavior modification, and pharmacologic therapy with fluoxetine and gabapentin. Within 6 weeks, the family reported moderate improvement, with marked and sustained improvement following dose adjustments and continued behavior modification. By 14 weeks, aggressive incidents had substantially decreased, and after 1 year of therapy, bite behavior had resolved. Long‐term follow‐up demonstrated stable improvement, improved tolerance of veterinary handling through cooperative care training, and restoration of household safety and quality of life. This case highlights the importance of early recognition, comprehensive assessment, and timely use of combined behavioral and pharmacologic interventions in juvenile dogs with severe aggression, emphasizing that young age should not preclude appropriate treatment.

## 1. Introduction

Aggression and anxiety‐related behaviors in juvenile dogs represent a significant welfare concern and are a leading cause of relinquishment and behavioral euthanasia in dogs under 3 years of age. Genetics, early developmental environment, social exposure, learning history, and early medical experiences all play critical roles in shaping emotional regulation, impulse control, and social behavior. Puppies removed prematurely from the dam, raised with inadequate social exposure, or bred without behavioral selection are at increased risk for fear, anxiety, impulsivity, and aggression later in life.

Early‐onset aggression may manifest as growling, snapping, biting, or intense overarousal as early as 6–8 weeks of age. These behaviors are frequently mischaracterized as normal “puppy behavior,” leading to delayed intervention. When untreated, early aggression can escalate in frequency and severity, resulting in breakdown of the human–animal bond and increased risk of injury. Timely intervention—including environmental management, behavior modification, and, when indicated, psychopharmacologic support—can significantly improve outcomes, enhance learning, and preserve placement in the home.

This case report describes a juvenile Australian Shepherd with severe, escalating aggression beginning at 9 weeks of age, multiple bite incidents, and significant household safety concerns. The report highlights risk factors, diagnostic considerations, and the successful use of a multimodal treatment plan incorporating management, behavior modification, and combined pharmacologic therapy.

## 2. Case Presentation

### 2.1. Signalment and Presentation

A 9‐month‐old, 15‐kg spayed female Australian Shepherd was evaluated for escalating aggression, including multiple bite incidents toward both familiar and unfamiliar people.

### 2.2. History

The dog was purchased at 8 weeks of age from a private sport‐performance breeder. She was one of a litter of seven puppies (four females and three males). All puppies were removed from the dam at 6 weeks of age, and the dam was returned to a co‐owner. The litter was raised with minimal social interaction; the puppies were kept in an outdoor kennel, did not interact with other dogs, and had restricted contact with humans aside from weekly visits by potential purchasers.

Initially, the patient appeared friendly and engaged with all family members. The household consisted of a married couple and their adult daughter, all with prior experience with dogs, including Australian Shepherds. The family′s goal was a companion capable of participating in occasional dog sports such as agility. No other animals were present in the home at acquisition.

Shortly after purchase, the puppy developed severe chronic diarrhea lasting approximately 6 weeks. Diagnostics included multiple veterinary visits, fecal testing, radiographs, and laboratory analysis, ultimately resulting in a diagnosis of *Giardia*. Treatment included deworming medications (metronidazole followed by fenbendazole; dosages unknown), subcutaneous fluids, and dietary trials. Gastrointestinal signs resolved completely.

Repeated free‐catch urinalyses were performed due to submissive urination when approached or greeted. All results were unremarkable, and the behavior resolved by 20 weeks of age. The dog was easily house‐trained and had no further urinary concerns.

At 9 weeks of age, the puppy growled when the female guardian attempted to pick up a food toy near her. From that point onward, aggressive behaviors—including growling, lunging, snapping, grabbing, holding, and biting—were observed in multiple contexts: during play, when excited, during handling, and when people approached food puzzle toys. These behaviors were directed toward all family members, visitors, and veterinary staff. Frequency and intensity escalated over time.

The family received repeated reassurance from the breeder, trainers, friends, and the primary veterinarian that the behavior was normal puppy energy, mouthiness, or due to insufficient leadership. Despite this, biting continued and resulted in a severe wrist sprain to the female guardian, multiple puncture wounds (some requiring medical treatment), and extensive bruising (Level 5, Dr. Ian Dunbar bite scale).

Over time, only the male guardian handled the dog, as the female family members became fearful. Aggression toward visitors included barking, jumping, grabbing, circling, and biting, particularly near the backyard pool. Similar behaviors occurred at the front door when people entered the home. As a result, the puppy was often confined, leashed, or separated during visits.

Outside the home, leash walking became increasingly difficult. By 5 months of age, the dog reacted aggressively toward strangers and other dogs. She attended puppy classes starting at 20 weeks and reportedly performed well with cues, showed no overt aggression in class, and continued into a juvenile class. However, she did not engage in social play, and only the male guardian attended classes.

At 6 months of age, the puppy immediately charged and bit an adult visitor (the family′s son), causing a puncture wound (Level 3, Dr. Ian Dunbar scale). Aggression continued in contexts involving food, toys, handling, visitors, car rides, and exposure to people or dogs through windows. Other behaviors included barking, panting, pacing, and lunging at passersby.

The family used verbal and physical corrections and frequent crate time‐outs (up to 15 min). No aversive equipment was used. The dog underwent ovariohysterectomy at 7 months without complications. A private trainer worked with the family for 4 weeks but noted extreme overarousal and aggression, requiring leash or protected contact.

By 9 months of age, the dog spent most of her time confined in a crate or bedroom. She did not display separation‐related distress, noise sensitivities, prey drive, or compulsive behaviors [1].

### 2.3. Physical Examination and Diagnostics

At the time of ovariohysterectomy, CBC and serum chemistry were within reference ranges. At the behavioral consultation, CBC, chemistry, and preprandial and postprandial bile acids were all normal. Neurologic and ophthalmic exams were unremarkable. No behavior‐modifying medications had been used prior to referral.

During the initial consultation, the dog was in good physical condition (BCS 5/9) with no evidence of pain, sensory deficits, allergies, or ongoing gastrointestinal disease [2]. She displayed moderate anxiety in the clinic, characterized by pacing, panting, restlessness, and hypervigilance, and remained close to the male guardian throughout the visit. The patient did not explore the room or engage in playful behaviors.

### 2.4. Diagnostic Assessment

Based on the history and examination, the following diagnoses were considered [3–5]:

• Impulse‐control aggression
• Possessive aggression (food, toys, and puzzles)
• Fear‐ and anxiety‐related aggression
• Socialization deficits
• Generalized anxiety disorder

### 2.5. Treatment

At presentation, household dynamics were severely compromised, with two family members unwilling to interact with the dog. Rehoming and behavioral euthanasia had been discussed prior to referral. A decision was made to pursue treatment with frequent reassessment [6, 7]. Although the dog had been exhibiting abnormal behaviors since she was 9 weeks, no psychopharmacological medications had been prescribed prior to the initial behavior consultation at 9 months. The reason given for this was that their general veterinarian considered the patient too young for behavioral medications [8]. The family was given no other pharmacological intervention options prior to their behavior consultation [9].

A comprehensive plan was implemented, including safety management, environmental modification, behavior modification, and medication according to the most recent published behavior guidelines by AVSAB and AAHA.

Extensive management and safety plans were outlined to protect the welfare of the patient and the human members of the household [10–12]. Management included keeping this patient on a well‐fitted leash and harness system within the home and documenting in a logbook all triggers to overarousal and overreactivity or aggression. A head collar was not used due to a history of aggression with close handling around the head and face, and a basket muzzle was not initially recommended for the same reasons [13, 14]. Gates, crates, and x‐pen fencing were used within the home to allow the female members of the house to feel safer while passing through rooms, to provide a safe buffer zone, and to comfortably sit in the same room as the patient. The clients were asked to discontinue all punishment or correction‐based interactions, and to instead employ positive reinforcement including whispered praise and small food treats for calm behaviors [10, 11]. Any forms of rough play were strongly discouraged.

Behavior modification protocols were introduced, including teaching the patient to relax in a variety of situations (Dr. Karen Overall′s Relaxation Protocol), and to self‐regulate with mindful breathing exercises using Dr. Karen Overall′s “take a breath” protocol [15]. These protocols lead to more predictable and consistent interactions and can be a critical component of a behavior plan to help the patient learn alternative behaviors to replace inappropriate responses. In‐home training sessions took place every other week for 16 weeks with a certified positive trainer (CCPDT, PPG, and VSPDT). Videos were submitted to the authors at monthly intervals to reassess progress and offer guidance to next steps. Refresher training sessions took place as needed for the following year. Positive punishment, including verbal correction, was discontinued because this could lead to increased anxiety, frustration, and aggression [14, 16, 17].

The family was encouraged to positively reinforce the patient for all relaxed behaviors, such as asking for sit or settle behaviors prior to interacting. New calming routines were introduced into the household including playing soft music and settling quietly on a bed or mat at specific times of day such as midmorning, midafternoon, and between dinner and bedtime [18, 19]. All food‐based puzzle toys were to be given only when the patient was in her crate, behind a gate, or within her x‐pen fencing for safety reasons. The patient was completely removed from the crate or room prior to retrieval of food‐based puzzle toys to ensure family safety [20]. Walks through the neighborhood were limited to off‐peak hours to avoid other people and dogs, and the dog was taken to local private‐reserved play yards on weekends. The patient was kept inside the house during the family′s pool activities to minimize overarousal and was eventually taught to relax on a mat around the pool deck while others were swimming.

Pharmacologic treatment was initiated with fluoxetine, a serotonin reuptake inhibitor, at 0.6 mg/kg PO q 24 h (recommended dose: 1–2 mg/kg PO q 24 h) and gabapentin, a calcium channel delta 2 ligand blocker, at 13 mg/kg PO q 12 h (recommended dose 20–30 mg/kg PO q 12 h), with plans for titration. Treatment doses initially fell below standard dosing ranges due to family concerns such as anorexia, lethargy, and “drugging their puppy,” having been warned by their breeder and family veterinarian that behavior medications would “be dangerous” and would “make things worse” for their puppy [9, 21–25]. The low‐starting doses were chosen to maximize owner compliance and minimize initial side effect concerns in this young dog. Gabapentin was selected as an adjunct for its anxiolytic and inhibitory effects on excitatory neurotransmission [15, 26–29]. Findings demonstrated associations between high aggression or impulsivity and low concentrations of serotonin [9, 22]. In several studies, dogs have responded favorably to the administration of fluoxetine in the treatment of a variety of aggression and anxieties [9, 22]. The synergistic benefit observed with combined pharmacologic treatment and behavioral intervention likely reflects posttranslational modulation of intracellular signaling pathways, particularly enhanced efficiency of cAMP‐dependent cascades. These effects promote activation of downstream protein kinases and increased expression of neurotrophic factors such as BDNF, thereby facilitating synaptic plasticity, activity‐dependent protein synthesis, and learning processes that potentiate behavioral modification [1]. Medication selection and dosing were explained in detail, including mechanisms of action and evidence for use in canine anxiety and aggression.

### 2.6. Follow‐Up and Outcome

At the 6‐week follow‐up, the family noted no medication side effects and a subjective 25% reduction in aggression and reactivity (no C‐BARQ used). Medication doses were increased with fluoxetine adjusted to 20 mg PO once daily (1.3 mg/kg) and gabapentin increased to 300 mg PO (20 mg/kg) twice daily, and further behavior modification guidance was provided [30–32].

At the next recheck, approximately 14 weeks after treatment initiation, the family reported approximately 75% improvement (subjective assessment by family members). Aggressive behaviors toward food, visitors, and during walks had improved with careful management and behavior modification. Overarousal decreased, and no further bite incidents had occurred since the previous visit. Rough play and confrontational interactions were further discouraged during the re‐evaluation, and more positive games were suggested, such as playing with a flirt pole involving a carefully practiced play‐pause‐play routine, where calm behavior was reinforced with brief moments of more active play. The family was also taught to teach drop‐it games without confrontation using a happy phrase such as “Oh, what do you have?” to encourage a fast drop‐it response in exchange for a small food treat [30]. Cooperative care training and veterinary visit preparation were introduced [6]. The use of preveterinary medications was recommended including combinations of trazodone (5–10 mg/kg), alprazolam (0.03 mg/kg), and increased gabapentin from her daily dose to a higher event‐specific anxiolytic dose (in this patient′s case, from 300 mg twice daily as an adjunctive maintenance med to a dose of 600 mg or 40 mg/kg) to be given 2–3 h before an appointment at the discretion of the referring general practitioner [31, 32] [60].

At annual follow‐up, the patient exhibited stable, long‐term improvement with no aggressive incidents, improved veterinary handling, successful muzzle training, and continued maintenance on fluoxetine (1.3 mg/kg PO once daily) and gabapentin (15 mg/kg PO twice daily) without adverse effects. The family chose to maintain all behavior medications.

## 3. Discussion

Genetics, early social exposure, and learning history play substantial roles in the development of anxiety and aggression in dogs [33–39]. In this case, both the dam and sire were later found to have exhibited aggressive behaviors yet continued to be bred for physical traits [37]. Early removal from the dam, limited social exposure, and breeding for high‐intensity sport traits likely contributed to the patient′s presentation. It may also be important to note that early, prolonged illness such as *Giardia* with chronic diarrhea constitutes significant physiological stress during critical periods of development. Repeated activation of the hypothalamic–pituitary–adrenal (HPA) axis from this early stress can lead to altered cortisol regulation and HPA set‐point changes. Theseneuroendocrine alterations may impact brain development and stress responsivity, increasing vulnerability to later neuropsychiatric and somatic disorders [60].

Fear and anxiety can manifest as aggression through autonomic nervous system responses [35, 40, 41]. Generalized anxiety lowers thresholds for reactivity and impulse control, increasing the likelihood of aggressive responses. Impulse‐control aggression is often explosive and contextually linked to perceived control or frustration [42–51]. The patient′s positive behavior in training classes at 20 weeks of age could be a result of her favorable relationship with the male handler, or from potentially being overwhelmed and “shut down” in the presence of unfamiliar people and dogs in an enclosed training space.

Food‐ and possession‐related aggression emerged early in this case and escalated over time, likely influenced by early developmental stressors. Correction‐based handling and rough play possibly exacerbated arousal and aggression [14, 18] [58]. Early intervention with combined behavior modification and pharmacologic treatment was critical. Fluoxetine improved serotonergic modulation of fear, arousal, and impulse control, whereas gabapentin provided anxiolytic and inhibitory effects on excitatory pathways [9, 15, 21–29] [59].

Early intervention is critical. This case demonstrates that psychopharmacologic treatment should not be withheld solely due to young age. Combined medication and behavior modification improved learning capacity, reduced arousal, and restored household safety. Although the improvement scores in this paper were subjective opinions given by the family, they noticed an overall improvement in behavior and were happy with the patient′s progress. Fluoxetine and gabapentin were effective, well tolerated, and instrumental in this patient′s successful outcome.

This case underscores the importance of early recognition, proactive treatment, breeder accountability, and the integration of medication with behavior modification to prevent escalation of juvenile aggression and preserve the human–animal bond. Ongoing management and long‐term medication will help to maintain all progress for this patient.

## Author Contributions

Lynn Honeckman: conceptualization, methodology, writing—original draft preparation, writing—review and editing.

## Funding

No funding was received for this manuscript.

## Consent

Written informed consent was obtained from the owner for publication of this case report. This case was exempt from institutional ethics approval as part of routine clinical practice.

## Conflicts of Interest

The author declares no conflicts of interest.

## Data Availability

The data that support the findings of this study are available on request from the corresponding author. The data are not publicly available due to privacy or ethical restrictions.
